# Artificial Intelligence Support for Informal Patient Caregivers: A Systematic Review

**DOI:** 10.3390/bioengineering11050483

**Published:** 2024-05-12

**Authors:** Sahar Borna, Michael J. Maniaci, Clifton R. Haider, Cesar A. Gomez-Cabello, Sophia M. Pressman, Syed Ali Haider, Bart M. Demaerschalk, Jennifer B. Cowart, Antonio Jorge Forte

**Affiliations:** 1 Division of Plastic Surgery, Mayo Clinic, 4500 San Pablo Rd, Jacksonville, FL 32224, USA; 2 Division of Hospital Internal Medicine, Mayo Clinic, Jacksonville, FL 32224, USA; 3 Department of Physiology and Biomedical Engineering, Mayo Clinic, Rochester, MN 55905, USA; 4 Department of Neurology, Mayo Clinic College of Medicine and Science, Phoenix, AZ 85054, USA; 5 Center for Digital Health, Mayo Clinic, Rochester, MN 55905, USA

**Keywords:** artificial intelligence, machine learning, informal caregiver, quality of life, burnout, ambient intelligence, digital health

## Abstract

This study aims to explore how artificial intelligence can help ease the burden on caregivers, filling a gap in current research and healthcare practices due to the growing challenge of an aging population and increased reliance on informal caregivers. We conducted a search with Google Scholar, PubMed, Scopus, IEEE Xplore, and Web of Science, focusing on AI and caregiving. Our inclusion criteria were studies where AI supports informal caregivers, excluding those solely for data collection. Adhering to PRISMA 2020 guidelines, we eliminated duplicates and screened for relevance. From 947 initially identified articles, 10 met our criteria, focusing on AI’s role in aiding informal caregivers. These studies, conducted between 2012 and 2023, were globally distributed, with 80% employing machine learning. Validation methods varied, with Hold-Out being the most frequent. Metrics across studies revealed accuracies ranging from 71.60% to 99.33%. Specific methods, like SCUT in conjunction with NNs and LibSVM, showcased accuracy between 93.42% and 95.36% as well as F-measures spanning 93.30% to 95.41%. AUC values indicated model performance variability, ranging from 0.50 to 0.85 in select models. Our review highlights AI’s role in aiding informal caregivers, showing promising results despite different approaches. AI tools provide smart, adaptive support, improving caregivers’ effectiveness and well-being.

## 1. Introduction

More than 43 million family caregivers in the United States alone provide complex medical care to relatives, with nearly half performing nursing tasks, often learning independently [1,2]. Technology aimed at supporting these caregivers should incorporate personalized systems based on user personas. For example, the CarePortfolio platform in the TOPIC project serves as an information resource, emotional support, and communication tool between informal and formal caregivers, enhancing task management and scheduling [3]. Meanwhile, in countries like Italy with aging populations and caregiver shortages, initiatives like the GUARDIAN ecosystem offer robotic assistance and mobile applications to improve the daily lives and care quality of the elderly through human–robot interactions [4]. Moreover, artificial intelligence (AI) has been proven to be beneficial in patient care, from facilitating communication for those with cerebral palsy using facial recognition-based Morse codes to preventing pressure ulcers in immobile patients and detecting early signs of Alzheimer’s disease [5,6,7]. AI-driven wearable devices also enable remote patient monitoring [8]. Despite these advancements, the full potential of AI in enhancing informal caregiver support remains largely untapped.

### 1.1. Background

The aging global population has led to a surge in the demand for informal caregivers. Currently, millions of caregivers tend to family members with chronic and life-limiting conditions. In the United States alone, over 43 million family caregivers play a crucial role in supporting the healthcare system [1,9,10]. 

An informal caregiver is generally a close friend or family member who offers consistent support to a patient. While the contributions of these caregivers are invaluable, the responsibility of caring for a loved one, often coupled with a lack of choice in assuming the caregiver role, can impose significant stress and burdens, with female carers experiencing particular strain [11,12,13,14].

Caregivers confront a range of challenging situations, from managing the pain of terminally ill patients to overseeing at-home rehabilitation post-stroke [15,16]. Despite these hurdles, a mere 7.3% of them receive training for their crucial role [9]. It is important to integrate caregiver assessment and management into every patient’s care process [11]. This involves regular evaluations for conditions like post-traumatic stress disorder [17], the provision of digital tools aimed at bridging the knowledge gap [18,19,20], and offering enhanced support [3,21,22,23,24,25,26].

Artificial intelligence (AI) empowers machines to perform tasks that typically require human intelligence, such as logical reasoning, learning, and problem solving [27]. In healthcare, the rapid advancement of innovative solutions like digital healthcare management, and particularly those driven by AI, addresses both longstanding and emerging challenges [28]. Algorithms from AI subsets, like machine learning (ML) and natural language processing (NLP), have consistently complemented various facets of patient care [29,30]. When combined with human input, AI enhances precision and efficiency, with ML, in particular, aiding in pattern recognition, decision making, and facilitating personalized interventions [30]. 

Various AI solutions, including an array of support robots [31,32,33,34], have been developed to address the need for assistance in care. AI-enabled devices have the potential to significantly enhance the independence of older adults in managing their health. This technological support not only promotes autonomy and self-reliance among the elderly but also helps in reducing the burden on caregivers [35]. This is achieved through facilitating coordinated care and analyzing data from various facets of a patient’s life and care, taking into account their frailty [36]. Such data encompass aspects like sleep quality [37] as well as seniors’ mobility, cognitive functions, and communication abilities [38]; however, these tools predominantly serve patients or professional caregivers in hospitals and nursing centers [39,40,41], or solely collect patient data through smart homes and ambient assisted living, offering little to no guidance or information to informal caregivers [42,43,44]. These tools are crucial in caregiving, significantly easing the burden on caregivers; however, they primarily focus on the routine and daily care of elderly individuals [45,46], rather than addressing the specific needs of patients. This distinction is important, as the care required for a patient can vastly differ from that for a healthy elderly person or child, and addressing these unique needs can greatly impact the lives of caregivers. Research focusing on aiding caregivers of patients with diverse needs remains limited. These studies primarily investigate the application of AI to directly support informal caregivers, rather than indirectly through patient care. Methodologies vary from evaluating burnout risk among informal caregivers of patients undergoing dialysis or suffering from amyotrophic lateral sclerosis (ALS) to enhancing their quality of life [47,48,49], as well as providing caregiving knowledge [50]. AI assistance may include robotic aid [51] and initiatives aimed at reducing the caregiving burden by improving caregivers’ work–life balance [52]; however, challenges such as ethical concerns over invasions of privacy due to data recording, the risk of system malfunctions, or providing inaccurate information, may arise. Furthermore, caregivers might become excessively dependent on these AI services [53].

### 1.2. Problem Statement and Research Questions

The majority of research focusing on AI aid for informal caregivers primarily targets those caring for patients with Alzheimer’s and dementia (35–37), neglecting the unique assistance needs of caregivers for patients with other chronic diseases. As the need for long-term care grows daily and informal caregivers play a pivotal role, we explored AI-driven solutions addressing informal caregiver burdens beyond those of dementia and Alzheimer’s patients. 

Our systematic review aims to answer the following questions:

1. How does AI impact the support of informal caregivers for patients?
2. How well do various AI strategies perform in caregiver-related tasks for informal caregivers?
3. What obstacles prohibit the integration of AI solutions in caregiving?
4. What weaknesses exist in the present research, and which areas are recommended for future exploration?

## 2. Methods

In this section, we detail the structured methodologies applied to identify and analyze the relevant literature on the use of artificial intelligence in assisting informal caregivers. Section 2.1. outlines the comprehensive search strings, including most related search terms and keywords, and describes our search methods across multiple databases. We then proceed with the study eligibility and selection process in Section 2.2., providing our PRISMA flow diagram. The quality of the studies is evaluated in Section 2.3. Finally, we conclude with a data synthesis and analysis in Section 2.4.

### 2.1. Databases Searched and Search Strategy

We combined research terms and keywords from different units using Boolean operators—AND, OR, and NOT—to refine our literature search. ‘AND’ was used to narrow results to documents containing all specified terms, enhancing search specificity. ‘OR’ broadened the search to include documents with any terms, increasing coverage. ‘NOT’ excluded irrelevant terms, streamlining the dataset for a more focused analysis. Table 1: Two independent investigators conducted a systematic search across four electronic databases and the Google Scholar search engine to identify relevant studies on artificial intelligence’s role in supporting informal caregivers. The selected databases were PubMed, Scopus, IEEE Xplore, and Web of Science. We conducted our search on 1 September 2023, without any date restrictions. The search string was tailored to suit the unique features of each database. For detailed search strings per database, see Supplemental File S1.

### 2.2. Study Eligibility and Selection Process

We included studies that utilized AI-based tools or algorithms to assist informal caregivers of patients.

We excluded studies if AI’s primary function was solely data collection from patients (e.g., sensors, ambient assisted living (AAL)), they were without empirical data involving human subjects (e.g., literature reviews, book reviews, commentaries), they did not involve family or informal caregivers as participants, they mainly centered on diagnosing/screening dementia patients or aiding caregivers of Alzheimer’s disease patients, their investigations were not centered on AI technology, they were focused on caregivers tending to healthy individuals, articles were not available in English, or we could not retrieve the full texts.

**Figure 1:**
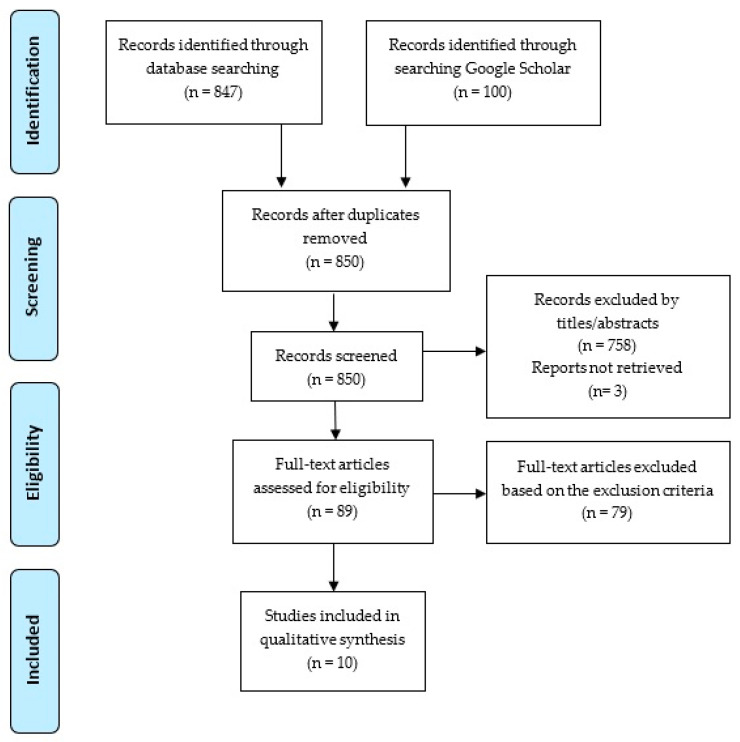
PRISMA flow diagram. Study selection process.

We structured our review based on the Preferred Reporting Items for Systematic Reviews and Meta-Analyses (PRISMA) 2020 guidelines [54] (Figure 1). After searching the mentioned databases using our search string, we systematically reviewed each paper’s sections for completeness. Articles were included if they matched our search criteria or contained relevant substrings. Duplicates were removed using Endnote software (version 20.4.1).

### 2.3. Data Quality and Risk of Bias Assessment

We employed a three-step approach to assess the quality and relevance of articles. Initially, titles and abstracts were screened for relevance. Subsequently, articles were skimmed for pertinent details before a thorough assessment was made in relation to our research questions. Given the diverse nature of AI methodologies in caregiving and the absence of standardized reporting, instead of traditional risk of bias tools we crafted a tailored approach. Each study was critically appraised by the first two authors, focusing on its design, AI methodology clarity, and validation techniques. In cases of disagreement, the third author made the final determination. Emphasis was placed on studies with transparent reporting, and we consulted domain experts to ensure depth of understanding. Potential biases inherent to our review process are acknowledged in the limitations section.

### 2.4. Data Synthesis and Analysis

We extracted key details from the selected studies aligned with our review focus, including author names, publication year, and study location.

## 3. Results

### Characteristics of Included Studies

Our initial search produced 947 articles. After applying the eligibility criteria, 10 articles remained, discussing the use of artificial intelligence to support informal caregivers of patients.

The studies in the reviewed articles were conducted from 2012 to 2023 across various countries, including the Netherlands [53], Thailand [55], Italy [47], Canada [56], Germany [50,52], Ireland [48,49], Korea [51], and United States [57].

Eight of these studies primarily employed machine learning models [47,48,49,50,51,52,55,57], one utilized a chatbot [56], and another incorporated model-based reasoning [53]. Additionally, one study developed a novel health risk analysis system, based on a risk analysis classifier (RAC) and using the SCUT algorithm [55]. Regarding validation methods, four studies [47,49,50,57] implemented Hold-Out validation, one solely used 10-fold CV [48], and another deployed both 2-fold CV and 10-fold CV [52]. It is noteworthy that two studies did not specify their validation methods [51,56].

In studies using SCUT with neural networks (NNs) and LibSVM, accuracy ranged from 93.42% to 95.36%. Global accuracy varied between 71.60% and 78.70%, while Caregiver Quality of Life Index-Revised (CQLI-R) metrics demonstrated 68% to 92% accuracy based on categories like physical and social characteristics. Metrics from different datasets showed accuracy between 80.00% and 99.33%. Precision for SCUT (NNs and LibSVM) was between 94.81% and 95.95%. CQLI-R metrics’ precision varied from 45% to 92%, and certain dataset metrics approached near-perfect precision scores (0.9993). Recall values for SCUT (NNs and LibSVM) spanned 93.42% to 95.40%. For CQLI-R metrics, the range was 45% to 92%. The F-measure in SCUT (NNs and LibSVM) studies was between 93.30% and 95.41%. CQLI-R metrics had F-measures from 52% to 84%. Area under the curve (AUC) values showcased model variability. In quality of life (QoL) and clinical decision support system (CDSS) models, AUC ranged from 0.50 to 0.85. Other metrics displayed AUC values from 0.72 to 0.83. Table 2 shows the characteristics of the included studies.

## 4. Discussion

Although the volume of data on AI-powered tools that assist informal caregivers and family members of patients is limited, there is growing research in this area, which is the focus of our study. We analyzed the extracted data from these studies, categorizing and discussing them in terms of how AI supports mental health, enhances decision making, and reduces burdens to improve quality of life for caregivers. This comprehensive approach enables us to clearly define the diverse roles that AI plays in caregiving contexts.

### 4.1. Implications and Key Findings

Our systematic review reveals that AI holds significant promise in aiding informal caregivers of patients across various dimensions. Research has evidenced a range of metric scores, contingent on different contexts. Notably, AUC is a key metric, providing a comprehensive assessment of a classifier’s ability to distinguish effectively between positive and negative classes. Additionally, accuracy reflects the model’s overall correctness, with values closer to 100% indicating higher reliability. Some studies have explored the development of an ambient agent to aid caregivers of depression patients, utilizing model-based reasoning [53]. Meanwhile, other research has introduced advanced systems for evaluating caregivers’ health risks across mental, physical, and social domains, offering tailored interventions. Notably, a study achieved a maximum accuracy of 95.36% and a precision of 95.95% in the social domain when using a neural network [55].

In evaluating burnout risk among caregivers of dialysis patients and creating a stress measurement tool, one study reported a global accuracy of 78.70% and an AUC of 80.2% using their test sample for a deep neural network (DNN) model [47]. Using chatbots, one study examined providing customized, immediate emotional support to family caregivers at a non-profit organization [56]. Another personalized system provided tailored educational content to caregivers. Their artificial neural network (ANN) model achieved a low training error (MSE: 8.585 × 10^−8^) and demonstrated consistent performance on the validation set (MSE: 7.731 × 10^−8^), indicating no overfitting [50]. In 2020 a study [48] focused on caregivers’ burdens using random forest algorithms and achieved the best AUC in their M2 group, indicating better classification capabilities. In the next year, the same group assessed the QoL of the caregivers, and the full model for predictors achieved the best metrics across F1 score, recall, precision, and AUC [49]. The F1 score is a comprehensive metric that considers both precision and recall. Recall (sensitivity) and precision show the fraction of correctly identified positives and the correctness of the positive predictions, respectively. The *p*-value and T-value are metrics of statistical significance. In a report by [51] on “Dori”, a robot assisting elderly individuals, they reported a T-value of 0.96 and a *p*-value of 0.342 for caregiver management, and a T-value of 0.01 with a *p*-value of 0.990 for medication instruction. This suggests both groups—caregivers and medical staff—view these aspects similarly, with minimal differences in their ratings. In [57], ML effectively analyzed spoken conversations to assess caregivers’ anxiety and quality of life. The Generalized Anxiety Disorder 7-item (GAD) metric showed the highest recall at 92%, and the overall CQLI-R had an accuracy and specificity of 76% and 73%, respectively. The most recent study, from 2023, used an ML algorithm to assist family caregivers in making healthcare decisions and executing caregiving tasks. For the datasets of sizes 100, 500, and 1000, the model achieved accuracies of 80%, 99.33%, and 99.33% respectively, with corresponding F1-scores and 10-fold CV results improving proportionally.

#### 4.1.1. Mental Health Support for Family and Informal Caregivers

While taking care of loved ones can place a significant burden on patients’ family members, the mental health of caregivers is often overlooked.

Joerin et al. [56] attempted to address this gap by using “Tess” as a low-cost, user-friendly support system for various types of caregivers, especially the family members of patients. Tess is a proprietary psychological AI-powered on-demand chatbot introduced by X2AI Inc. (X2) (San Francisco, CA, USA). It can be accessed via text messaging or integrated with voice-enabled Amazon systems. While Tess previously demonstrated a reduction in symptoms of anxiety and depression in students by 18% and 13%, respectively [58], it was integrated with the “Elizz Caregiver” program at SE Health in Canada to assess its suitability for caregivers’ needs in two phases. Tess exchanged 12,000 messages with caregivers, and 88% of users found it helpful.

Offering a range of support, from cognitive behavioral to psychodynamic therapy, chatbots such as Tess present affordable and flexible programming. This adaptability is crucial when considering aids for caregivers. Furthermore, the absence of a control group in the study might introduce bias, complicating determinations about the program’s true effectiveness. It is worth mentioning that while digital solutions offer many advantages, it is crucial to acknowledge their inherent limitations. Greater familiarity with such tools often correlates with increased usage.

Studies show that many family caregivers face health issues due to insufficient exercise, neglecting annual health check-ups, and experiencing sleep disturbances [55]. Utilizing web-derived health data, Suksawatchon et al. [55] developed the health risk analysis system (HRAS). This system processes caregiver information collected by nurses, transforming it to evaluate health risks in mental, physical, and social domains. Based on these evaluations, the HRAS suggests interventions to aid family caregivers of disabled individuals. Notably, their use of NNs yielded an accuracy rate exceeding 90% across all datasets. In another study by Aziz et al. [53], based on [59], an ambient support for caregivers of patients with depression was developed and evaluated. They paid attention to three important aspects of caregiving, these being incoming stressors, mediating conditions, and caregiver outcomes. They examined a dynamic model focused on interactions during caregiving under stressful conditions, and through computational modeling and simulation they illustrated the impact of support or lack thereof on both caregivers and the individuals they care for; however, the specificity of this computational model to a particular scenario raises concerns about its generalizability to diverse contexts. Moreover, without benchmarking against a recognized gold standard or empirical data, the veracity and validity of the results remain uncertain.

#### 4.1.2. How Does AI Enhance Decision Making for Caregivers?

Machine learning offers assistance to overwhelmed caregivers by providing a decision-support system that guides them toward the most appropriate next steps in care. In a newly published study [52], researchers implemented a machine learning-based digital case manager designed to assist family caregivers in balancing their caregiving responsibilities with their personal lives. Data were collected through a questionnaire completed by caregivers. The algorithm was trained using seven distinct modules that outlined various use cases and their corresponding schemas. Furthermore, labels indicating subsequent steps were incorporated to guide caregiving actions. During the evaluation, their obtained model’s performance significantly improved in metrics when moving from Dataset 100 to Dataset 500/1000 in two-fold and ten-fold cross-validation (two-fold CV 56% to 99.7% and ten-fold CV 73% to 99.8%). While the study employed the random forest classifier as its model, employing more advanced and complex algorithms, like deep learning techniques, could potentially yield better results; however, these sophisticated models require more comprehensive data and usually do not perform well with limited datasets, such as the ones utilized in this study. This limitation may compromise the reliability and generalizability of the outcomes.

This is the same as in [50], which used a shallow artificial neural network that possessed a wide architecture. In comparison with methods like random forests, this neural network can efficiently leverage parallel processing. With the appropriate training strategy, it also managed to avoid overfitting.

Since informal carers often lack the formal nursing training required to care for their loved ones optimally, Wolff et al. [50] tackled the knowledge gap in informal caregivers through a personalized educational system. While their prior model utilized a statistical ranking to prioritize topics, they enhanced it by integrating a learning mechanism to factor in user feedback. They trained this updated system on 3200 artificially generated profiles and assessed its accuracy using another 640 profiles, specifically examining the system’s topic ordering ability. Although their model can adapt based on real caregiver feedback, its limited training data and specialized setup may constrain its generalizability, despite decreasing training and validation errors.

Kim et al. [51] developed an adult-guided, caregiver-monitored robot named Dori to support older adults, while also addressing ethical concerns and incorporating feedback from stakeholders. Operating within the field of the human-centered artificial intelligence (HCAI) framework, the authors tailored the programming of Dori, drawing from preferences identified in caregiver groups from previous studies. The robot offers services with effective design features such as search functions, medication instructions, cognitive and physical activity prompts, and caregiver management tools. Additionally, it provides affective design services like emotional support; however, the robot has not been tested in a long-term or real-world setting, which can decrease the generalizability of the obtained model.

#### 4.1.3. AI, Caregivers’ Burden, and Quality of Life

Informal caregiving often affects caregivers’ QoL, particularly impacting their mental and physical well-being, especially when caring for patients with both motor and cognitive issues. To devise an effective support system for caregivers, it is crucial to understand the challenges they face and the underlying factors [60,61,62]. In a recent study, Antoniadi et al. [49] examined the QoL of caregivers for patients with amyotrophic lateral sclerosis (ALS). Their prior research [48] identified caregivers’ quality of life and psychological distress as primary indicators of burden. This observation was quantified using a random forest model as a predictor that demonstrated a sensitivity of 0.92 and specificity of 0.78. Building on these findings, they further explored factors affecting caregiver QoL and designed a machine learning alert system for clinical decision making support. Utilizing the McGill QoL questionnaire, they preprocessed their data by handling missing values and transforming variables. They initially employed a logistic regression model and later advanced to the more powerful XGBoost for predictive modeling. This integrated approach aims to enhance clinical decision making and interventions, potentially providing caregivers with timely support and resources. Additionally, their utilization of SHAP (SHapley Additive exPlanations) amplifies model explainability and feature importance analysis in machine learning. It is worth noting that the inherently subjective nature of assessing QoL, combined with the rarity of ALS and the subsequent small sample size, can introduce challenges and potential prediction errors.

In a separate study focused on identifying burnout risk factors among caregivers of patients with chronic degenerative diseases, particularly chronic kidney disease (CKD), researchers [47] employed validated questionnaires. Following a principal component analysis (PCA) with varimax rotation on 29 questionnaire items, they utilized the resulting information as input variables for developing a neural network model. The model was applied to four key components: emotional and physical impact, assistance burden, need for interaction with medical professionals, and perceived tension.

In the test group, the predictions achieved 72% accuracy when caregivers were not experiencing stress and 83.7% accuracy when they were, resulting in an overall accuracy rate of 78.7%. Notably, the study identified care load and tension as the most significant factors in predicting caregiver stress.

The NN model they developed has the potential to be incorporated into a mobile app, which could alleviate the burden on caregivers while also reducing caregiving costs and saving time. The same can be said about the study by Demiris et al. [57], which demonstrated that, by utilizing ML classifiers, one can accurately predict QoL improvements of caregivers of hospice patients. For the first time, their AI models employ a custom-trained automated speech recognition system using DeepSpeech2 to transcribe speech, and a logistic-regression-based classifier to utilize both transcribed text and extracted acoustic features to predict caregivers’ quality of life and anxiety levels. By integrating sound dimensions, they achieved a precision of 92% and an accuracy of 89%.

**Figure 2:**
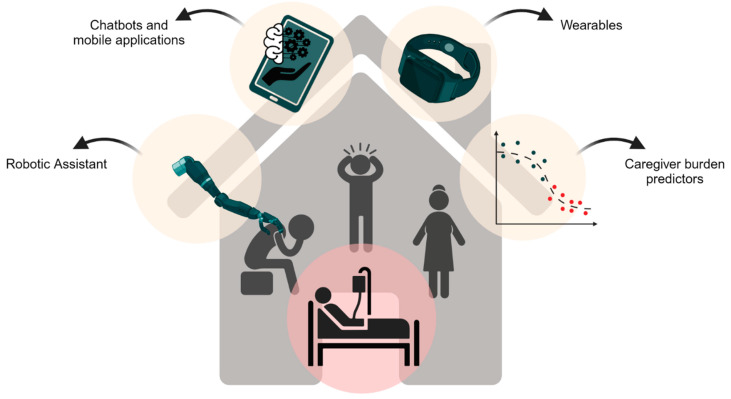
AI can assist informal caregivers through various means, including robots, chatbots, mobile applications, wearables, and tools that predict caregiver burden.

Leveraging classical ML models is advantageous with smaller datasets, enabling the real-time evaluation of new data; however, their inability to process extensive datasets limits their generalizability. Alternatively, employing transfer learning enhances the accuracy and generalizability of small datasets by utilizing DNNs, offering a viable solution for achieving superior results [57]. In the field of artificial intelligence and machine learning model deployment, the integrity, consistency, and methodology of data preprocessing hold extensive importance in shaping outcomes. Equally crucial is the selection of a suitable target population. For instance, research indicates that younger caregivers often face a more pronounced decline in QoL [49]. Requirements based on the demographics of caregivers, such as age, can significantly influence the effectiveness of the chosen model. This, in turn, can impact the precision of the instrument and alter clinical decision-making hierarchies. Such variations can be attributed to differing life aspirations and engagements, underscoring the need to factor in these elements when exploring AI’s role in the caregiving landscape. Figure 2 illustrates a spectrum of AI technologies that are enhancing the support system for informal caregivers. These tools, including robots, chatbots, mobile applications, wearables, and predictive tools for caregiver burden, are each tailored to address specific aspects of caregiving. Collectively, they contribute to a more supportive and manageable caregiving experience [63].

### 4.2. Limitations

Although the studies highlight the high quality and utility of AI-powered tools in reducing the burden on informal caregivers, several challenges affect their daily implementation. Caregivers may become overly dependent on these tools, potentially diminishing direct interaction with patients. Additionally, there are privacy concerns, as these devices collect sensitive information to offer appropriate assistance, and the risk of inaccuracies due to algorithms being trained on limited datasets. This underscores the need for more diverse caregiving scenarios to address various challenges [48,49,53].

Web-based tools, such as [55], face accessibility issues related to Internet connectivity. Moreover, both staff and caregivers require adequate training to utilize these tools effectively, interpret results, and manage complex care directives, which can be time-consuming and intricate [48,49,50,52,55]. Similarly, mobile applications demand a certain level of technological literacy from caregivers, which may not always be feasible [47]. Furthermore, the training of these tools on hypothetical rather than real-world cases may compromise their accuracy when applied in actual caregiving settings. Consequently, these tools cannot replace comprehensive care management and often necessitate significant external supervision [52].

In this systematic review, we encompassed studies from various settings and methodologies. The diversity in reported results and data across these studies precluded a consistent assessment of bias risk, potentially neglecting inherent biases in the studies. Additionally, due to these discrepancies, we refrained from comparing the derived algorithms. While PROBAST [64] is a widely recognized tool for assessing the risk of bias in prediction model studies, its application to our selection of studies was challenging due to the variety of AI methodologies and the broad context in which they were used. As a result, a consistent risk of bias assessment using PROBAST was not feasible. Instead, we aimed to critically discuss the methodologies and results of each study, emphasizing their individual limitations and strengths. Due to the abundance of research on family caregivers for dementia and Alzheimer’s patients, we excluded related studies, potentially introducing a bias into our assessment. Future reviews could benefit from a broader scope, including caregivers for different conditions. While this review incorporated some studies based on models that exhibit AI-like decision-making capabilities, some of these models might not align with contemporary AI paradigms due to their publication era. It is essential for future research to consider integrating state-of-the-art AI models, enhancing the accuracy and precision of the algorithms, thus optimizing decision-making processes.

### 4.3. Recommendations for Future Research

Future research can expand the application of these models across diverse healthcare settings, particularly in decision-making domains. Implementing these programs in real-world scenarios and varied settings can provide a broader spectrum of caregivers. It is essential to recognize that, for example, the support required by caregivers of paralyzed patients might differ significantly from those tending to patients with major depressive disorder; thus, tailoring solutions to specific caregiving situations is crucial. Furthermore, increasing the sample size and employing more advanced AI algorithms can enhance the accuracy of predictions and reduce the potential for bias in forecasting. In future systematic reviews, it is advisable to consider conducting a meta-analysis to reduce the risk of bias and yield more robust results.

## 5. Conclusions

In our in-depth analysis of 10 studies exploring the role of AI in supporting informal caregivers, we examined the strengths and limitations of each methodology. These models consistently produced impressive results, with AUC values ranging from 0.72 to 0.83 and accuracies between 71.60% and 78.70%. Our findings highlight the significant contribution of informal caregivers in patient care; however, considering their immense burden and reduced quality of life, there is an urgent need for enhanced support for this population. Leveraging AI-driven models can provide intelligent, efficient, and tailored assistance to these caregivers.

## Figures and Tables

**Table 1:** Research units and search terms.

Research Unit	Search Terms/Keywords
**Technologies**	AI, ML, NLP, artificial intelligence, machine learning, deep learning, neural networks, and natural language processing
**Care context**	home caregivers, home care, care at home, family caregivers, home caretaker, home carers, home patient caregivers, home-based patient care, home caregivers for patients, home care for patients, non-professional caregiver, informal caregiver, unpaid caregiver, unpaid informal carer, and relative caregiver
**Exclusion criteria**	healthy child, general child care, nurse, doctor, physician, and medical professional

**Table 2:** Included studies’ characteristics.

Author	Location	Study Aim	Study Design	Participants	Type of AI	Validation Method	Metric Scores
Aziz et al., 2012 [53]	Netherlands	Designing an ambient agent to assist caregivers of patients with depression.	Model development and validation	Three fictional types of caregivers (CG1, CG2, and CG3) in simulation experiments	Model-based reasoning	Simulation experiments Verification of identified properties	N/A
Suksawatchon et al., 2018 [55]	Thailand	Introducing a new expert system to assess caregivers’ health risk levels in mental, physical, and social domains and provide customized interventions for each.	Model development and validation	Data of 150 caregivers to train and evaluate the RAC model	RAC: HRAS (classifier technique and rule-based classifier) SCUT algorithm (hybrid sampling technique) ML: Decision tree Naive Bayes (Kernel) NN LibSVM	k-fold CV Experts with annotated and unseen data	Accuracy with SCUT: NN: mental (ACC at 94.71%), social (ACC at 95.36%); LibSVM: physical (ACC at 93.42%) Precision with SCUT: NN: mental (94.81%), physical (94.81%), and social (95.95%) Recall with SCUT: NN: mental (94.74%); LibSVM: physical (93.42%); NN: social (95.40%) F-measure with SCUT: NN: mental (94.67%); LibSVM: physical (93.30%); NN: social (95.41%)
Costa et al., 2018 [47]	Italy	Assessing burnout risk in dialysis patient caregivers and develop a stress measurement tool.	Model development and validation	Seven hundred and thirteen family caregivers of dialysis patients	ML: DNN	Hold-Out validation	Sample training (%) and sample test (%): Correct forecast (no stress): 62.80, 72.00 Correct forecast (stress): 78.80, 83.70 Global accuracy: 71.60, 78.70 Area under ROC: 0.802, 0.802
Joerin et al., 2018 [56]	Canada	Examining how a mental health chatbot provides customized, immediate emotional support to family caregivers at a non-profit organization.	Technical report	Relatives of patients aged 20–59, with most between 50 and 59	Tess chatbot	N/A	N/A
Wolff et al., 2019 [50]	Germany	Using a personalized system to offer targeted educational content to caregivers based on their needs.	Model development and validation	Three thousand and two hundred artificially created profiles for training the ANN and six hundred and forty randomly generated profiles for the validation set	ML: ANN	Hold-Out Validation End-Validation Set Utilized Termination Criterion	Total training epochs: 374,700 Incorrectly ordered training profiles: 8 out of 3200 Final MSE (training set): 8.585 × 10^−8^ Final MSE (validation set): 7.731 × 10^−8^
Antoniadi et al., 2020 [48]	Ireland	Predicting caregiver burden in ALS patients and identify related features using machine learning.	Model development and validation	Ninety ALS patients and their primary caregivers	ML: random forest	10-fold CV	Metric/model: Model M2, Model M3, and Model M9 Ten-fold CV—sensitivity: 0.82, 0.80, and 0.71 Ten-fold CV—specificity: 0.77, 0.83, and 0.63 Independent test data—sensitivity: 0.92, 0.80, and 0.84 Independent test data—specificity: 0.78, 0.78, and 0.72 AUC: 0.85, 0.83, and 0.79
Antoniadi et al., 2021 [49]	Ireland	Identifying caregiver QoL predictors and creating models for a CDSS.	Model development and validation	Ninety patient and caregiver pairs	ML: LASSO XGBoost	Hold-Out Validation	Model: F1, recall, precision, and AUC Predictors of QoL: Baseline: 0.76, 0.72, 0.81, and 0.72; Full: 0.84, 0.83, 0.86, and 0.80; M7: 0.83, 0.83, 0.83, and 0.77 CDSS models: Baseline-CDSS: 0.52, 0.45, 0.62, and 0.50; Full-CDSS: 0.71, 0.72, 0.70, and 0.61; M10-CDSS: 0.75, 0.79, 0.72, and 0.65; M6-CDSS: 0.70, 0.72, 0.68, and 0.58
Kim et al., 2022 [51]	Korea	Developing “Dori,” a robot for supporting frail elderly at home, balancing their dignity and caregiver values within the HCAI framework.	Technical report	Caregivers, nurses, and clinicians	ML	N/A	Caregivers, medical staff, T-value, and *p*-value Cognitive activity: 6.05 (±1.77), 5.64 (±2.23), 0.96, and 0.344 Emotional activity: 6.36 (±1.78), 5.6 (±3.04), 1.63, and 0.109 Physical activity: 5.82 (±2.69), 5.32 (±2.62), 1.02, and 0.311 Medication instruction: 6.05 (±2.59), 6.04 (±1.64), 0.01, and 0.990 Caregiver management: 5.86 (±2.94), 5.4 (±2.32), 0.96, and 0.342
Demiris et al., 2022 [57]	United States	Evaluating ML classifiers’ relation to anxiety and QoL based on spoken words and features from caregiver–therapist talks.	Model development and validation	Dataset of 124 audio-recorded conversations between hospice patient caregivers and a therapist	ML: LR (text and audio) DL: (ASR System (DeepSpeech2))	Hold-Out Validation	Classifier: precision, recall, accuracy, and specificity CQLI-R (total): 73%, 79%, 76%, and 73% Physical: 80%, 86%, 83%, and 80% Financial: 69%, 90%, 81%, and 75% Social: 82%, 69%, 73%, and 78% Emotional: 77%, 63%, 68%, and 75% GAD: 92%, 88%, and 89%
Wunderlich et al., 2023 [52]	Germany	Using ML to guide family caregivers in healthcare decisions and caregiving tasks.	Model development and validation	Twenty-eight use cases, crafted by care experts	ML: random forest	Two-fold and ten-fold CV	Metrics: Dataset 100, Dataset 500, Dataset 1000 Accuracy: 80.00%, 99.33%, and 99.33% F1-score: 0.9306, 0.9993, and 0.9993 Two-fold CV: 56%, 97.78%, and 99.7% Ten-fold CV: 73%, 99.2%, and 99.8% Hamming loss: 0.01764, 0.00039, and 0.00039 Coverage error: 6.06, 4.85, and 4.94 Label ranking average precision: 0.9209, 0.9993, and 0.9993 Label ranking loss: 0.0648, 0.0011, and 0.0011

Abbreviations: ACC (accuracy), ANN (artificial neural network), ASR (automated speech recognition), AUC (area under the curve), CDSS (clinical decision support system), CQLI-R (Caregiver Quality of Life Index-Revised), CV (cross-validation), DL (deep learning), DNN (deep neural network), final MSE (final mean squared error), GAD-7 (Generalized Anxiety Disorder Scale, 7-item), HRAS (health risk analysis system), LASSO (least absolute shrinkage and selection operator), LR (logistic regression), ML (machine learning), NN (neural network), RAC (risk analysis classifier), and XGBoost (extreme gradient boosting).

## Supplementary Materials

The following supporting information can be downloaded at: https://www.mdpi.com/article/10.3390/bioengineering11050483/s1, Supplemental File S1: The detailed search strings per database.
