# Supplementary material for: Artificial Intelligence Support for Informal Patient Caregivers: A Systematic Review
Source: Bioengineering (Basel). 2024 May 12;11(5):483. doi: 10.3390/bioengineering11050483 (PMC11118398; doi:10.3390/bioengineering11050483)
Supplement: Supplementary file 1 [file bioengineering-11-00483-s001.zip › bioengineering-2971573-supplementary.pdf]

Supplemental File S1

01.09

Google scholar: first 100 out of 1380

(("artificial intelligence" OR "machine learning" OR "natural language processing") AND ("family caregiver" OR "informal caregiver" OR "unpaid informal carer") NOT ("healthy child" OR "general child care" OR "nurse" OR "doctor" OR "medical professional"))

PubMed: 282

((("Artificial Intelligence"[Mesh] OR "Machine Learning"[Mesh] OR "Natural Language Processing"[Mesh])) AND (("Caregivers"[Mesh] OR "Home Care Services"[Mesh] OR "family caregiver" OR "informal caregiver" OR "unpaid caregiver" OR "unpaid informal carer"[tiab:~0] OR "home care for patients"[tiab:~0] OR "Home Nursing"[Mesh] OR "home caretaker"[tiab:~0] OR "home carers" OR "home patient caregivers"[tiab:~0] OR "home-based patient care"))) NOT (("healthy child" OR "Nurses"[Mesh] OR "Physicians"[Mesh] OR "doctor" OR "medical professional" OR "professional caregiver" OR "nursing home"))

Scopus: 272

(ALL(("artificial intelligence" OR "machine learning" OR "natural language processing")) AND TITLE-ABS-KEY(("family caregiver" OR "informal caregiver" OR "unpaid informal carer")) AND NOT ALL(("healthy child" OR "general child care" OR "nurse" OR "doctor" OR "physician" OR "medical professional")))

IEEE: 142

(((((No Keywords Specified))) AND ("artificial intelligence" OR "machine learning" OR "natural language processing")) AND ("home caregivers" OR "home care" OR "care at home" OR "family caregivers" OR "home caretaker" OR "home carers" OR "home patient caregivers" OR "home-based patient care" OR "home caregivers for patients" OR "family caregivers" OR "home care for patients" OR "non-professional caregiver" OR "informal caregiver" OR "unpaid caregiver" OR "unpaid informal carer" OR "relative caregiver")) NOT ("healthy child" OR "general child care" OR "nurse" OR "doctor" OR "physician" OR "medical professional")

Web of Science: 151

1= ALL=("home caregivers" OR "home care" OR "care at home" OR "family caregivers" OR "home caretaker" OR "home carers" OR "home patient caregivers" OR "home-based patient care" OR "home caregivers for patients" OR "family caregivers" OR "home care for patients" OR "non-professional caregiver" OR "informal caregiver" OR "unpaid caregiver" OR "unpaid informal carer" OR "relative caregiver")

2= ALL=("artificial intelligence" OR "machine learning" OR "natural language processing")

3= ALL=("healthy child" OR "general child care" OR "nurse" OR "doctor" OR "physician" OR "medical professional")

4= #1 AND #2

5= (#4) NOT #3

("artificial intelligence" OR "machine learning" OR "deep learning" OR "neural networks" OR "natural language processing" OR "AI" OR "ML" OR "NLP") AND ("home caregivers" OR "home care" OR "care at home" OR "family caregivers" OR "home caretaker" OR "home carers" OR "home patient caregivers" OR "home-based patient care" OR "home caregivers for patients" OR "family caregivers" OR "home care for patients" OR "non-professional caregiver" OR "informal caregiver" OR "unpaid caregiver" OR "unpaid informal carer" OR "relative caregiver") NOT ("healthy child" OR "general child care" OR "nurse" OR "doctor" OR “physician” OR "medical professional")
